# Conformity and Mass Media Influence in the Sznajd Model on Regular Lattices

**DOI:** 10.3390/e26040307

**Published:** 2024-03-30

**Authors:** Maciej Wołoszyn

**Affiliations:** Faculty of Physics and Applied Computer Science, AGH University of Krakow, Al. A. Mickiewicza 30, 30-059 Krakow, Poland; maciej.woloszyn@agh.edu.pl

**Keywords:** opinion dynamics, sociophysics, phase transition, consensus, polarization, Sznajd model, agent based models

## Abstract

The polarization of opinions and difficulties in reaching a consensus are central problems of many modern societies. Understanding the dynamics governing those processes is, therefore, one of the main aims of sociophysics. In this work, the Sznajd model of opinion dynamics is investigated with Monte Carlo simulations performed on four different regular lattices: triangular, honeycomb, and square with von Neumann or Moore neighborhood. The main objective is to discuss the interplay of the probability of convincing (conformity) and mass media (external) influence and to provide the details of the possible phase transitions. The results indicate that, while stronger bonds and openness to discussion and argumentation may help in reaching a consensus, external influence becomes destructive at different levels depending on the lattice.

## 1. Introduction

Creating models and using them to understand, explain and predict the behavior of all types of systems is a basic tool of science. Sometimes those models are based on the expertise of one discipline that is applied and further developed in other fields. Sociophysics is a good example of such situations [1,2], and one of the most important and current issues in this area is modeling the dynamics of social opinion [3]. Since social systems are complex by their very nature, numerous models have been developed to grasp basic processes. Among the most commonly investigated are various versions of the voter model [4], Sznajd model [5], bounded confidence dynamics [6], majority rule model [7,8], Latané model of social impact [9,10], to name just a few. Researchers have addressed many important questions using these models in theoretical studies and computer simulations. Some recent developments have been made, for example, on the problem of consensus [11,12], divided communities and polarization [13,14,15,16,17], factors influencing the dynamics of opinion [18,19] (e.g., the so-called social temperature [20]), vanishing opinions [21], or the impact of mass media and advertising on the dynamics of opinion [22,23,24]. Various underlying lattices have been used [25], and behavior resembling phase transition (in a strict sense possible only in the thermodynamic limit) has received a lot of attention [26,27,28,29,30,31,32].

This study explores the Sznajd model of opinion dynamics applied to four different regular lattices. The basic version of this model is defined as a chain of actors (individuals) who have one of the two possible opinions, typically defined as +1 and −1 [5]. It is based on the psychological effect that two or more people who have the same opinion on a given issue are likely to convince others who interact with them (their neighbors). In the model, it comes down to a simple rule which states that if two adjacent actors have equal opinions, then they convince their neighbors (actors at adjacent sites of the chain). Since its introduction, many generalizations of the Sznajd model have been proposed: usage of various lattices or networks defining the structure of relations between the actors (square lattice, complete graph, or small-world networks), a parameter that specifies the probability of convincing (conformity), the introduction of several levels of opinion, see Reference [33] for a comprehensive review.

One of the proposed modifications has introduced the probability that opinion flips due to the external influence of mass media advertising [34,35]. When this probability is non-zero a complete consensus is not possible; the conditions for the number of actors having a given opinion changes abruptly when the network of relations is modeled with the regular square lattice. However, it has not been established yet whether this kind of phase transition also occurs on other lattices, and if it does, at what critical values of the relevant parameters. The first of these parameters is the probability of convincing (conformity) α related to the susceptibility of actors to the common opinion of pairs of other actors; for example α=1 means that an actor always adopts the opinion of a pair of agreeable actors after interaction with that pair. The remaining parameters determine the probability of the influence of the media related to the susceptibility of actors to such an external factor. In the case of two possible opinions, it is natural to expect that different media have access to the actors and try to advertise their agenda. The probability that the mass media convinces an actor to the +1 opinion is β, and generally, it can be different from the probability γ of convincing to the −1 opinion by other media.

This work addresses the issues discussed above for square lattices with von Neumann and Moore neighborhoods, triangular lattices, and hexagonal lattices. So far, it has also remained unclear how exactly both the probability of convincing other actors (neighbors) and the mass media influence the possibility of consensus. Therefore, the aim of this study is to provide a phase diagram answering that question as well.

## 2. Model and Methods

The version of the Sznajd model used in this work allows a pair of actors to convince others with probability 0<α≤1. All actors are also exposed to the external influence of mass media, advertising, etc. Here, for simplicity and following the discussion presented in Reference [34], it is assumed that both opinions are equally supported by the mass media, which means that only the symmetric case of β=γ is considered and the space of the considered parameters is reduced to (α,β).

**Figure 1:**
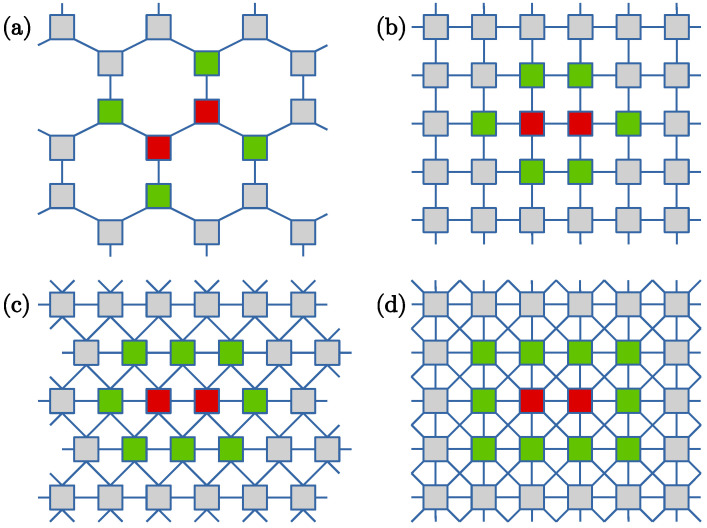
Lattices used in the simulations: (**a**) honeycomb (HC), (**b**) square with von Neumann neighborhood (SQ-VN), (**c**) triangular (TR), (**d**) square with Moore neighborhood (SQ-M). The red nodes illustrate examples of pairs of actors who try to convince their neighbors shown as the green nodes. In all cases periodic boundary conditions are used.

The opinion dynamics is modeled on four different regular lattices presented schematically in Figure 1. For each of them, periodic boundary conditions are applied, with equal numbers of rows and columns. The first is the honeycomb lattice (HC), with only $n_{NN}=3$ nearest neighbors of each actor. Figure 1a shows that for this lattice, any pair of actors (red color in the figure) has four neighbors (green) possibly influenced by the pair. The commonly used square lattice with the von Neumann neighborhood (SQ-VN) has $n_{NN}=4$ nearest neighbors of each actor. As illustrated in Figure 1b, a pair of actors can potentially influence six other actors. In the triangular lattice (TR), Figure 1c, $n_{NN}=6$ and each pair influences eight other actors. Finally, changing the neighborhood in the square lattice to Moore (SQ-M) increases $n_{NN}$ to eight and the number of neighbors of a pair of actors to ten, as shown in Figure 1d.

Each of the *N* actors located in the nodes of the used lattice has the opinion $s_{i}=+1$ or $s_{i}=-1$, which may, for example, correspond to being “in favor” or “against” some issue. The initial state of the system, $s_{i}\left(t=0\right)$ for all i=1,…,N is generated randomly. Each actor independently receives the opinion +1 with probability *p* and −1 with probability 1−p.

In one Monte Carlo step (MCS) of the dynamics simulation, the following sequence of operations is repeated *N* times:

1. An actor *i* is randomly chosen from i∈{1,…,N};
2. The actor’s opinion $s_{i}$ is exposed to the various mass media, some of them promoting +1 opinion, and some promoting the opposite opinion; in the considered symmetric case, it means that effectively $s_{i}$ is flipped to $-s_{i}$ with probability β;
3. An actor *j* is randomly chosen from the $n_{NN}$ nearest neighbors of the actor *i*;
4. If the opinions of the pair (i,j) are equal, $s_{i}=s_{j}$, then each of the neighbors of the pair is independently convinced of that common opinion with probability α.

After the completion of an MCS step, the average opinion *m* is calculated,

(1) $$m=\frac{1}{N}\sum_{i=1}^{N}s_{i}.$$

This asynchronous update scheme is repeated $t_{max}$ times, without stopping before reaching this time limit even in the case of reaching consensus when external influence is present (β>0), because such an influence may easily disturb the perfect consensus and the state of the system will then further evolve.

## 3. Results

**Figure 2:**
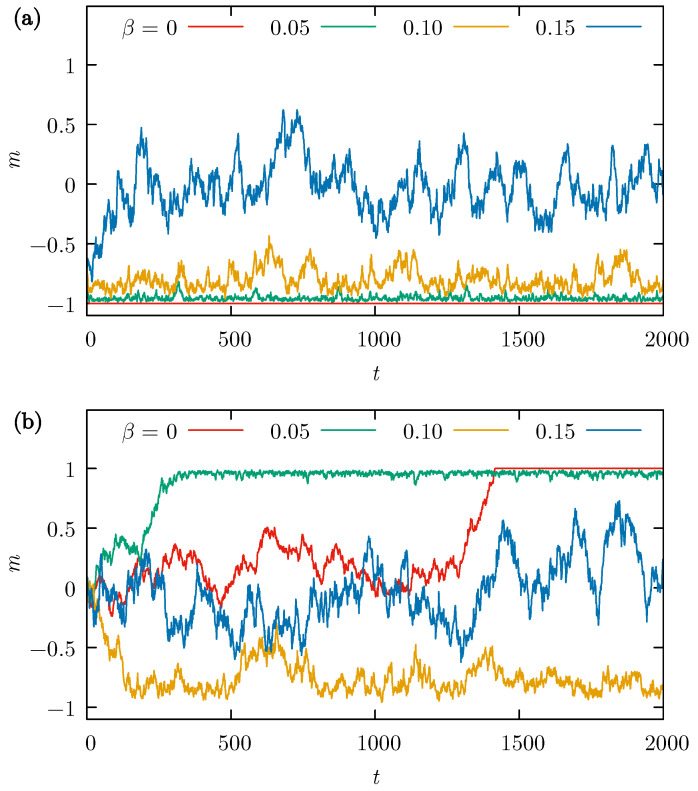
Examples of the time evolution of the average opinion *m*, with time *t* measured in MCS. Honeycomb (HC) lattice with N=1600 actors, probability of convincing the neighbors α = 1, and the external influence probabilities β=0, 0.05, 0.1, and 0.15. The initial state generated with two different probabilities *p* of $s_{i}\left(t=0\right)=+1$: (**a**) p=0.2 and (**b**) p=0.5.

The examples of the time evolution of opinions over 2000 MCS are shown in Figure 2 where the dynamics take place on the HC lattice, and an agreeable pair always imposes its opinion on the neighbors (α=1). Reaching full consensus, with all actors having the same opinion is clearly possible only when there is no external influence, that is, for β=0. Even a small β>0 does not allow such perfect agreement in the system; however, at lower values of β=0.05 or β=0.1, the vast majority of actors agree and the average opinion *m* is close to ±1.

The situation changes dramatically when the probability of external influence increases. Already at a value of β=0.15, the average opinion *m* oscillates around zero, indicating that the proportion of actors with both opinions is not very different and tends to fluctuate without a tendency to reach a state even close to what may be called consensus.

The difference between Figure 2a,b is that in the former p=0.2, while in the latter p=0.5. This difference in the initial condition means that in the first case, 20% of actors have the opinion +1, and the opinion of the remaining 80% is −1. In the second case, both opinions are represented in equal numbers, and reaching a consensus requires a much longer time as one of the opinions must gain a substantial majority first, which is an inevitable but long process. Since the unequal amounts of the opinions “+1” and “−1” in Figure 2a does not change the observed dynamic (in terms of the possibility of reaching a consensus) beyond the fact that one of the opinions is preferred as the final state, the value of p=0.2 is used in the calculations for simplicity of discussion of the average values of the opinions.

As mentioned above, the main objective of this paper is to discuss the interplay of conformity measured by the parameter α and the external influence β. To answer this question, simulations are performed over $t_{max}=10^{4}$ MCS, with the first 10% used for “thermalization”. The values of the average opinion *m* are further averaged over the remaining $t_{avg}=0.9t_{max}$ MCS, and then the mean value of such results obtained from R=48 independent simulations is calculated. The resulting quantity is denoted as 〈m〉 in the following discussion.

**Figure 3:**
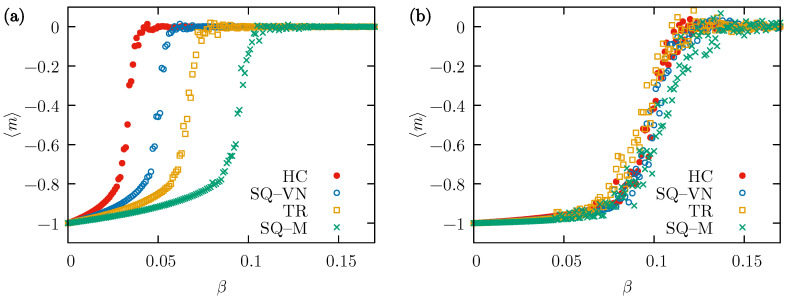
The mean value of the average opinion, 〈m〉, taken from R=48 simulations performed independently on all four types of lattices (HC, SQ-VN, TR, and SQ-M) with N=1600 actors. Calculated as a function of the external influence probability β for $t_{max}=10^{4}$ MCS and p=0.2. (**a**) The neighbors convincing probability α=0.15, and (**b**) α=1.

The results presented in Figure 3 explain the differences between the dynamics observed in different lattices when the conformity parameter α is fixed and the external influence varies. In Figure 3a, the average opinion for fixed α=0.15 changes when β increases. This abrupt change from a state of almost complete consensus to 〈m〉≈0 defines the critical value $\beta_{c}$ corresponding to the transition between the two states. Clearly, the amount of external influence needed to drive the system out of the consensus state increases with the number of neighbors (and the degree of the underlying graph) from $\beta_{c}\approx 0.03$ for the HC lattice to $\beta_{c}\approx 0.1$ for the SQ-M lattice. However, if α=1 as in Figure 3b, the differences between the lattices are very small and the answer to the external influence changes for all systems at approximately the same critical value of $\beta_{c}\approx 0.1$.

**Figure 4:**
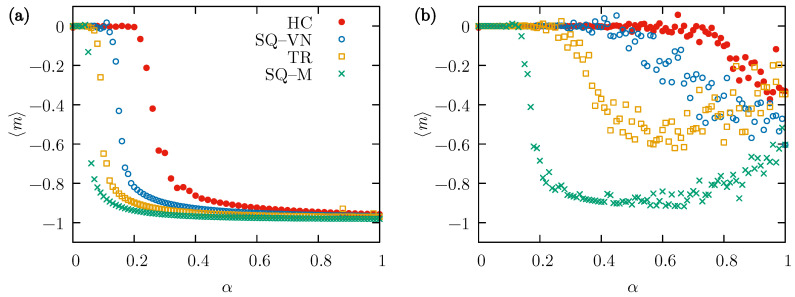
The mean value of the average opinion, 〈m〉, taken from R=48 simulations performed independently on all four types of lattices (HC, SQ-VN, TR, and SQ-M) with N=1600 actors. Calculated as a function of the neighbors convincing probability α for $t_{max}=10^{4}$ MCS and p=0.2. (**a**) The external influence probability β=0.05, and (**b**) β=0.1.

In contrast, when β is fixed at 0.05 and 〈m〉 is found as a function of the parameter α, a sudden transition to the consensus state is visible at the value of the parameter α which can be treated as the critical value $\alpha_{c}$ characteristic of this transition. That critical value decreases with the number of neighbors, as shown in Figure 4a. Similarly to the results of Figure 3a, the broadest range of the analyzed parameter values allowing for the consensus is observed for the SQ-M lattice, and the narrowest for the HC lattice. At a higher value of the probability of external influence, β=0.1, the situation is more complicated. It seems from Figure 3b that there is definitely a transition in the case of the SQ-M lattice, and probably only a certain majority of one of the opinions is reached at the final state in the three remaining lattices. The increase in 〈m〉 for the SQ-M and TR lattices visible in Figure 4b results from the fact that at higher values of α and at larger β, the evolution of the system sometimes reaches the final state corresponding to the “+1” opinion even for the used p=0.2.

## 4. Discussion

The results presented in the previous section indicate that a more organized approach is needed to obtain a detailed picture of the opinion dynamics on the analyzed lattices. One of the possible ways to address the main purpose of the work is to find the critical points $\left(\alpha_{c},\beta_{c}\right)$ that separate the observed states in the phase space of the parameters α and β. Revealing the position of the critical point with greater accuracy can be conducted by applying the finite-size scaling method based on the fourth-order Binder cumulant *K* defined as [36,37]

(2) $$K=1-\frac{\langle m^{4}\rangle }{3{\langle m^{2}\rangle }^{2}},$$

which is calculated for fixed values of α and β parameters, so that the cumulant is found as a function K(α,β).

In typical problems of statistical physics, for example, concerning the Ising model, it is used to detect the critical temperature and the type of phase transition. Continuous phase transitions are indicated by the change of *K* from 2/3 at low temperatures (T→0) to zero at the high-temperature limit (T→∞) [38]. At the critical temperature, the curves K(T) calculated for different system sizes cross, allowing an accurate numerical determination of the critical point [39,40].

**Figure 5:**
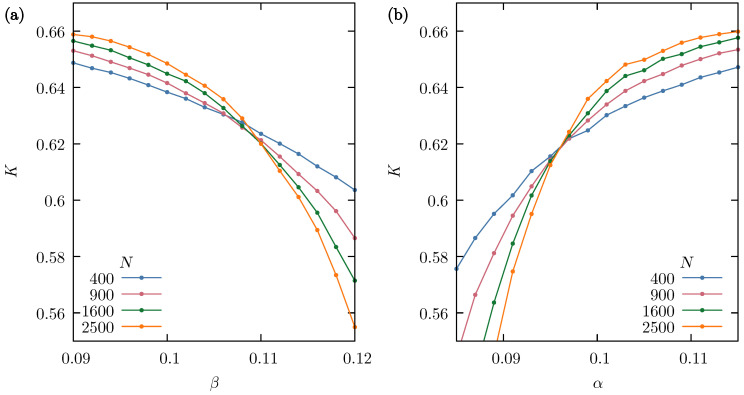
(**a**) Dependence of the fourth-order Binder cumulant K(α=1,β) on the external influence probability β for the square lattice with von Neumann neighborhood (SQ-VN) and the neighbors convincing probability α=1. (**b**) The fourth-order Binder cumulant K(α,β=0.05) as a function of the neighbors convincing probability α for the triangular lattice (TR) and the external influence probability β=0.05. In both cases, the results have been obtained for systems with size N=400, 900, 1600 and 2500 from R=48 calculations with p=0.2 and $t_{max}=10^{5}$ MCS.

For the Sznajd model in the version used in this work, the role of parameters α and β is analogous to temperature. It means that by finding *K* as a function of these parameters, it is possible to detect the critical points of the opinion dynamics. For better statistics and greater accuracy, longer simulation times ($t_{max}=10^{5}$ MCS) were used with a range of several different system sizes, N=400, 900, 1600 and 2500 actors. Figure 5a shows that the curves corresponding to the fourth-order Binder cumulants calculated for α=1 decrease from about 2/3 at small β and cross at the critical point $\beta_{c}\approx 0.11$. Therefore, the increased influence of the mass media is similar to the increase in temperature as a factor driving the system out of the ordered (consensus) state. The dependence of the cumulant on the neighbors convincing probability α is shown in Figure 5b for the fixed value of β=0.05. Again, the curves cross at one point, allowing one to find the critical value $\alpha_{c}\approx 0.96$. Yet, in this case, K→2/3 for increasing α, since more likely convincing helps to achieve consensus.

However, the results presented in Figure 5 provide only limited information about the interplay of two factors: external influence (mass media) and the ability of a pair of actors to convince their neighbors. For this reason, the procedure of finding the points where the curves of the fourth-order Binder cumulant K(α=const,β) cross was applied to the whole range of α∈[0;1], to find the critical points at which the planes K(α,β) calculated for different sizes *N* intersect. The applied procedure was based on finding the cross-section points of the pairs of fourth-order Binder cumulant curves calculated for different system sizes, and then averaging those results.

**Figure 6:**
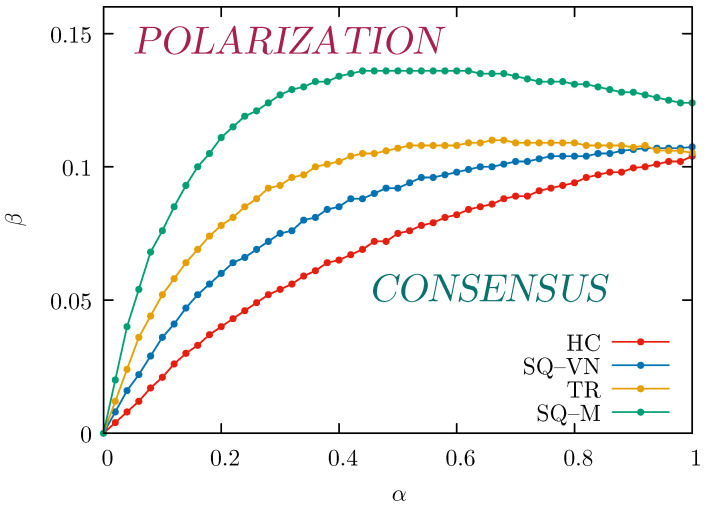
Phase diagram with points corresponding to the critical values $\left(\alpha_{c},\beta_{c}\right)$ separating the consensus phase (below a line) and the disordered phase (above a line) obtained for the four used lattices with numbers of actors between N=400 and N=1600 from R=48 simulations with p=0.2 and $t_{max}=10^{5}$ MCS.

The results of these calculations are presented in Figure 6. It shows the curves of the critical values $\left(\alpha_{c},\beta_{c}\right)$ that separate the ordered phase (consensus, at least partial) observed below those lines from the state when no consensus can be achieved and the opinions are polarized (above the lines). At α→0 also $\beta_{c}\to 0$, which confirms that when the chances of convincing others are very low, even minimal external influence destroys consensus. For all lattices, the critical values $\beta_{c}$ initially increase with α, demonstrating that the ability to find consensus is less prone to external influence if individuals are more likely to convince their neighbors. Interestingly, this growth is not only non-linear but in some cases limited only to α<0.65 (for the TR lattice) or α<0.5 (for the SQ-M lattice). It means that for the structures of social relations characterized by large numbers of neighbors, it is not only easier to convince large numbers of other actors but an additional effect is observed. That is, even with an unchanged external influence, the possibility of reaching a consensus is reduced, as the increased impact on others (larger α) means that it is also easier to spread the imposed external opinion, which in turn, promotes polarization.

The critical values $\beta_{c}$ are generally higher for lattices with a larger number of nearest neighbors, that is, $\beta_{c}^{\mathrm{HC}}\leq \beta_{c}^{\mathrm{SQ}-\mathrm{VN}}\leq \beta_{c}^{\mathrm{TR}}\leq \beta_{c}^{\mathrm{SQ}-M}$. However, as α increases, the differences between the lattices vary a lot. For example, at α=0.15 the critical values are $\beta_{c}^{\mathrm{HC}}\approx 0.032$ and $\beta_{c}^{\mathrm{SQ}-M}\approx 0.098$, which means that they differ by a factor of three; see also Figure 3a. At α=0.7 this factor is only 1.5 ($\beta_{c}^{\mathrm{HC}}\approx 0.090$ and $\beta_{c}^{\mathrm{SQ}-M}\approx 0.135$). Finally, if α→1, the differences between the values of $\beta_{c}$ are very small except for slightly larger $\beta_{c}^{\mathrm{SQ}-M}$, which is also visible in Figure 3b.

## 5. Conclusions

It is certainly no surprise that stronger interactions between the actors, in terms of the number of relations and neighbors, make it more difficult to prevent reaching a consensus when external influence, for example, in the form of mass media advertising is present. However, the results presented above reveal that the critical values of the mass media influence differ more or less between the considered lattices with various numbers of the nearest neighbors, depending on the probability of convincing those neighbors. The results reported here show that the differences between those critical values are the largest for the intermediate values of the convincing probability. Of course, it is not unexpected that very little convincing allows even small external influence to prevent reaching a consensus, independently of the type of lattice. At the maximum probability of convincing, the differences between the lattices are relatively small, only with the exception of the square lattice with the Moore neighborhood. Those differences are the largest at intermediate values of convincing probability. It is also interesting and somewhat surprising at first sight that for the triangular lattice and, in particular, for the square lattice with the Moore neighborhood, the largest chances of consensus are observed not for the largest probability of convincing the neighbors. This property might be due to the subtle interplay between the effect of convincing which is strengthened by a larger number of neighbors, while this larger number of neighbors also helps to spread the opinions induced by mass media.

Although the considered lattices do not exactly correspond to the real-world networks of interpersonal (or intergroup) relations, the differences between the results obtained in each of the cases reveal some factors important for opinion dynamics. The larger number of contacts limits social fragmentation and closing in social bubbles. This contributes to an increased ability to reach a consensus, certainly possible provided there is at least a moderate level of openness and trust in relationships with others which is the meaning of the parameter α used in this work (alternatively, one may argue, viewed as our naivety in adopting opinions of others, in this case pairs of our neighbors). On the other hand, the chances of polarization of opinion are greatly enhanced by stronger external influence (for example, due to aggressive strategies to find new readers, viewers, subscribers, followers, etc.), related to higher values of β in the current model. In fact, the results show that steering the opinion towards polarization can be as easy as providing enough external influence; for example, β>0.15 will have this effect for all levels of convincing probability α and no matter which of the lattices discussed here models the structure of relations. Alternatively, if the cost of such influence is too high, another possible strategy for driving the system towards polarization is to reduce α, for example, by reducing the exchange of information or trust; however, the effectiveness of this strategy depends on the type of lattice.

In summary, the relationship between the critical values of both considered factors was found for four different lattices. The results emphasize the importance of a detailed analysis of the conditions under which public opinion evolves. The data presented here indicate that while stronger bonds and openness to discussion and argumentation may help in reaching consensus, external influence becomes destructive even at relatively low levels, and the interplay between those two factors is rather complex. Therefore, future research should consider the potential effects of networks of realistic social interactions, including complete graphs, small-world, scale-free or growing networks, or even time-dependent large-scale complex networks [41] and many other factors, for example, anticonformity [42,43,44].

## Data Availability

Dataset available on request from the author.

## Abbreviations

The following abbreviations are used in this manuscript:

HC	Honeycomb lattice
NN	nearest neighbors
MCS	Monte Carlo step
SQ-VN	Square lattice with von Neumann neighborhood
SQ-M	Square lattice with Moore neighborhood
TR	Triangular lattice
